# Supplementary figures and images for: Transformation of Stimulus Correlations by the Retina
Source: PLoS Comput Biol. 2013 Dec 5;9(12):e1003344. doi: 10.1371/journal.pcbi.1003344 (PMC3854086; doi:10.1371/journal.pcbi.1003344)

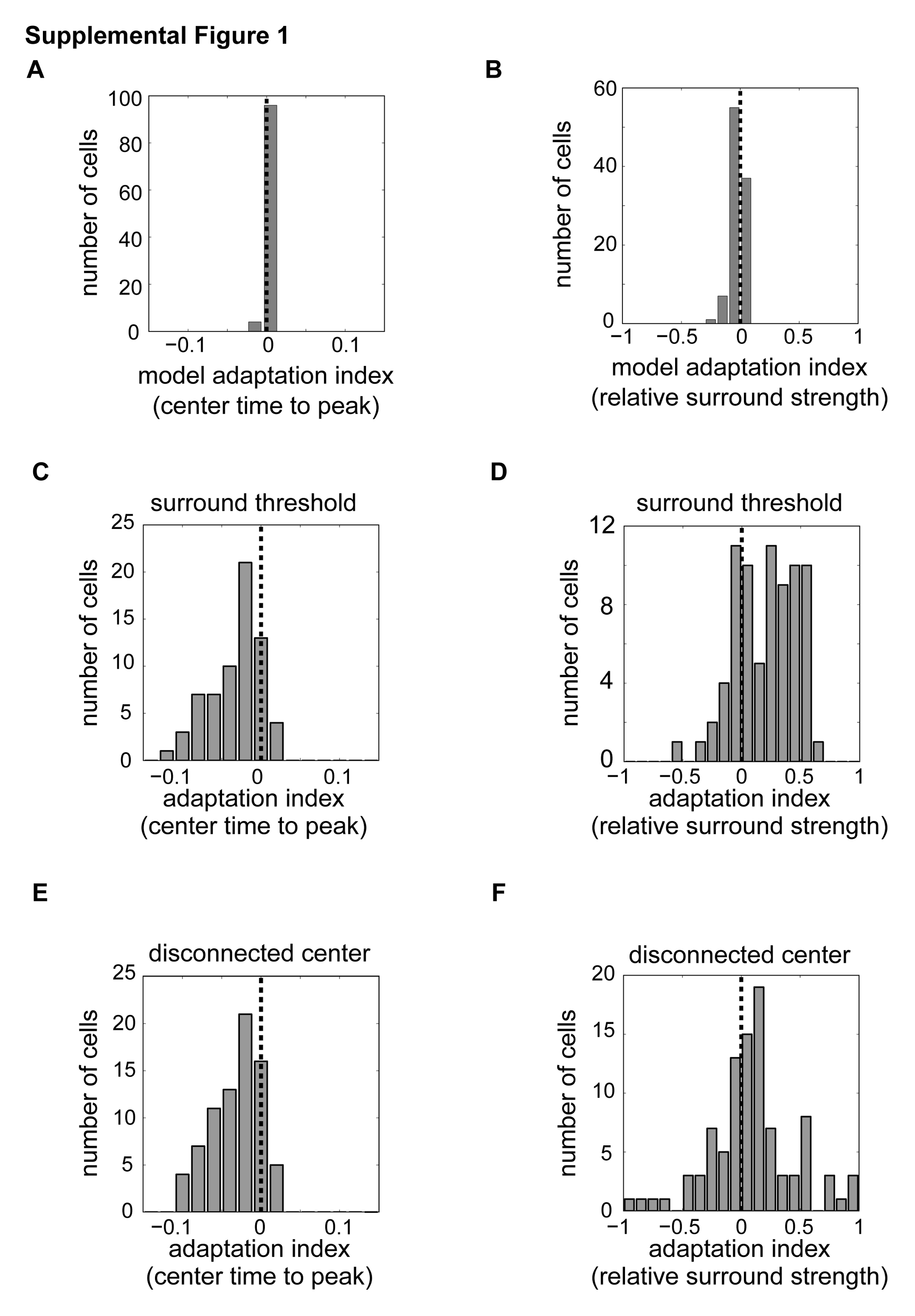

Supplement: Figure S1 — Receptive field results are validated with model neurons and are robust to analysis method changes. (A) Center time to peak for a population of non-adapting model neurons, plotted as in Figure 6A. Receptive fields were constructed as a difference of Gaussians. Surround radii (relative to center radii) had a mean of 2 and a standard deviation of 0.3. Surround weights (relative to center weights) had a mean of 1 and a standard deviation of 0.1. The model neurons do not show a large difference between stimuli in center time to peak. (B) Model neurons described in (A) show a slight bias toward smaller recovered relative surround strength under correlated noise compared to white noise. (C) Center time to peak is longer for white noise than for correlated noise when the surround only includes non-center pixels whose time courses are positively correlated with the time course of the peak negative pixel. (D) Relative surround strengths is marginally higher for correlated noise than for white noise under the same analysis as in (C). (E) Center time to peak is longer for white noise than for correlated noise when the center is not required to form a single contiguous component. The figure omits a few outliers that lie outside the range of the horizontal axis and have longer time courses under correlated noise. (F) Relative surround strengths is marginally higher for correlated noise than for white noise under the same analysis as in (E). (TIF) [file pcbi.1003344.s001.tif]

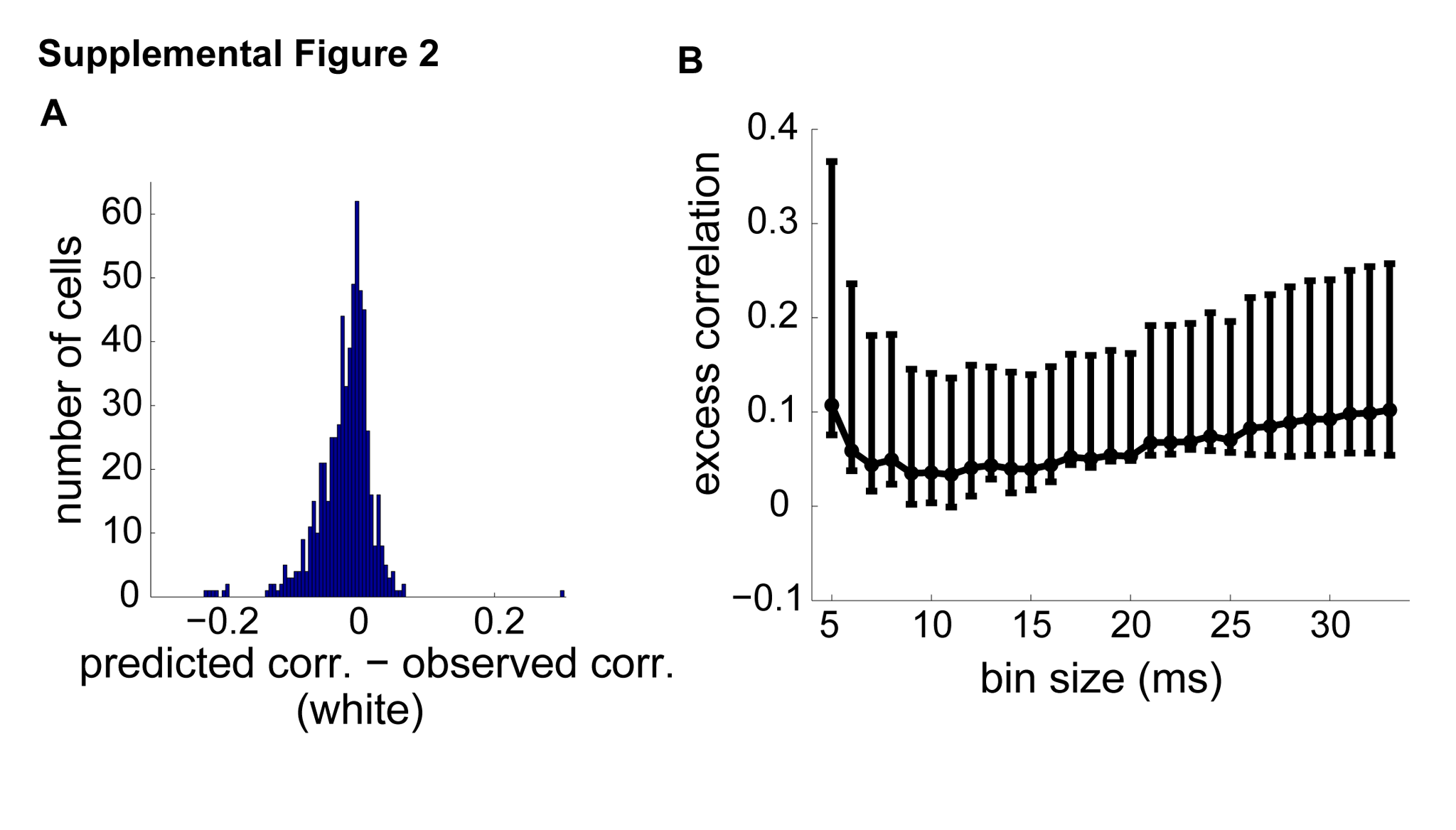

Supplement: Figure S2 — Correlation measurement controls. (A) As a control on the quality of the non-adapting LN model, we examined the difference between its predicted pairwise correlations under the white noise stimulus (the stimulus to which the model was fit) and the observed correlations. Since the model is a single-neuron model that does not attempt to capture pairwise correlations, we do not expect it to reproduce these correlations perfectly. Nevertheless, many cell pairs are well-predicted, indicating that their correlation is largely due to receptive field overlap. There is, however, a slight tendency for the model to underestimate correlations: this is likely due to its neglect of noise correlations. We note that such a bias will not effect the model's predicted excess correlation, unless it is very different in the two stimulus conditions. But such an effect would represent a form of non-trivial processing in its own right. (B) Our correlation measurements were based on binned spike trains. We measured excess correlation, in the spatiotemporal exponential dataset, for a variety of bin sizes. Its value is largely independent of bin size. Error bars represent bootstrap confidence intervals. All correlations reported in the main text were estimated from spike trains binned at . (TIF) [file pcbi.1003344.s002.tif]

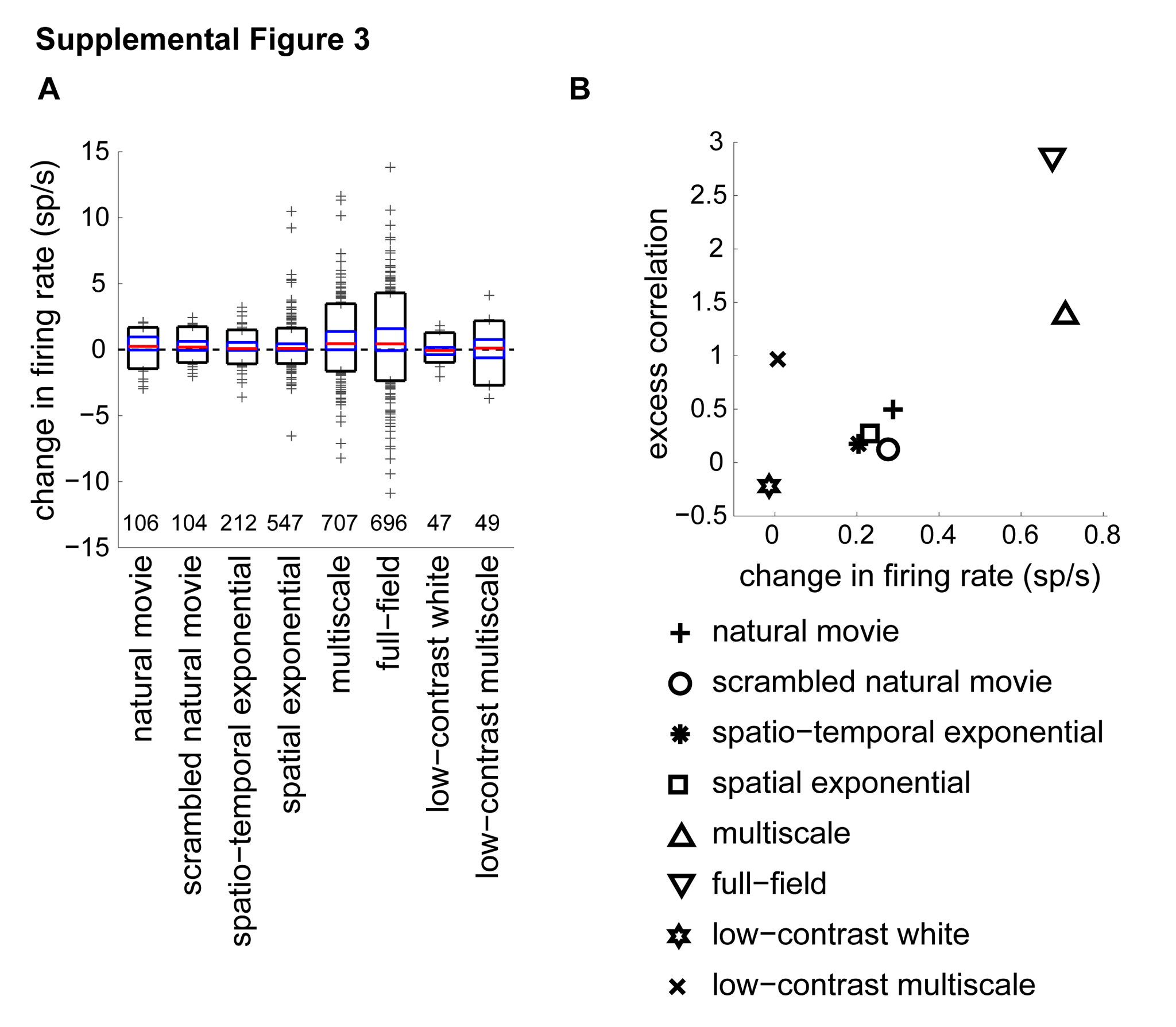

Supplement: Figure S3 — Firing rates are within a physiologically normal range. (A) Firing rates are similar across stimuli. Each cell's average white noise firing rate was subtracted to aid in comparison across experiments. Red line indicates the median for each stimulus. Blue boxes show the middle 50% of values; black boxes, the middle 90%. Gray crosses represent the outlying 10% of cells. Numbers below each box show the number of cells included. (B) Comparison of the excess correlation for each correlated stimulus to the average response (relative to the white noise response) shows that excess correlation is not simply a reflection of firing rate. (TIF) [file pcbi.1003344.s003.tif]

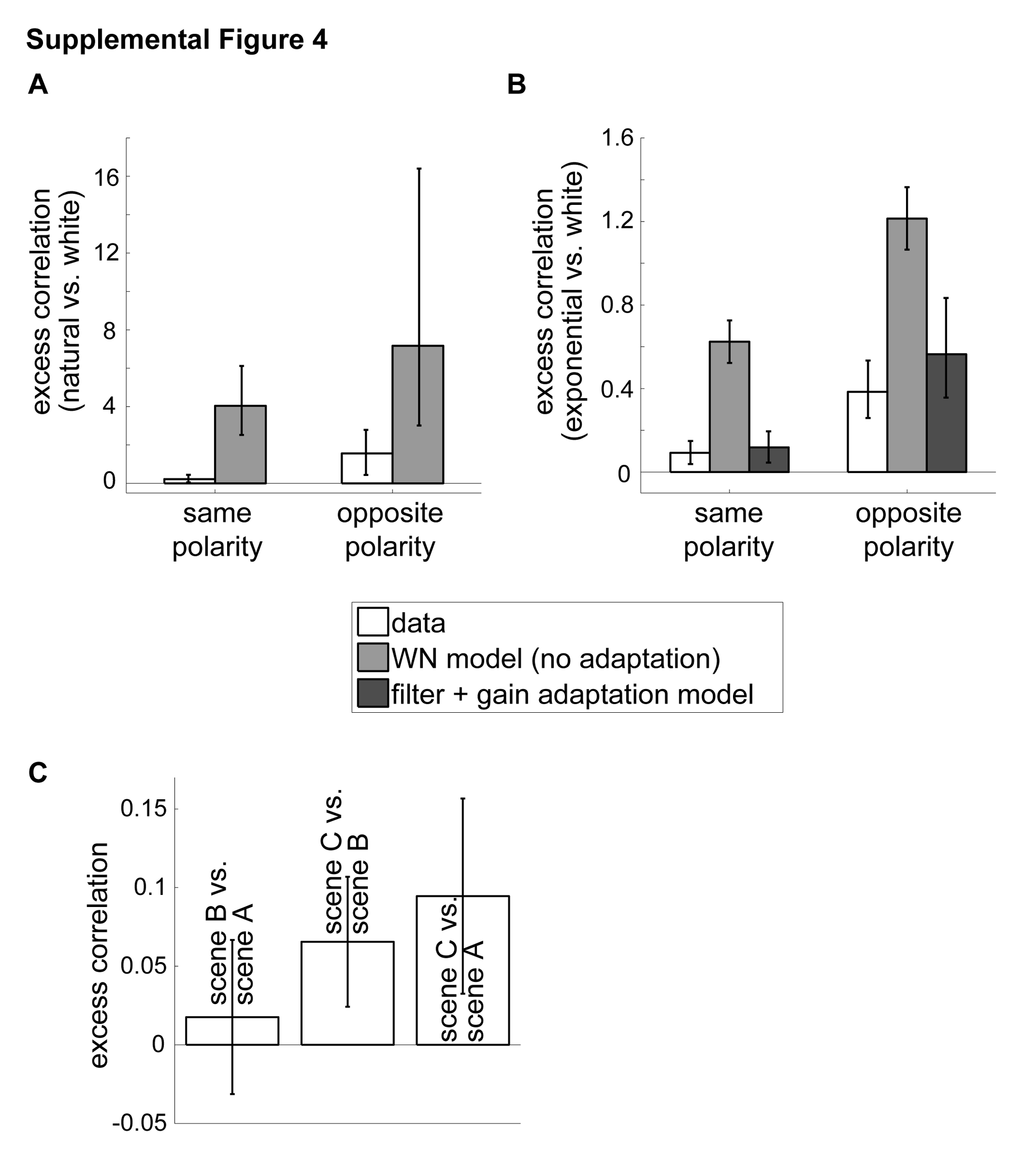

Supplement: Figure S4 — Dependence of excess correlation on response polarity and type of scene. (A, B) Excess correlation values for natural movie (A) and spatio-temporal exponential (B) stimuli separated by polarity of the recorded pair. Correlated stimuli evoked a greater increase in output correlations for ON-OFF pairs than for ON-ON and OFF-OFF pairs (white bars). This increase was consistent with correlation changes predicted by a non-adapting model for opposite-polarity pairs responding to natural scenes (A, rightmost bar) but was smaller than the expected correlation change in all other cases (gray bars). For the spatio-temporal exponential stimulus, where we could compute white noise and correlated noise receptive fields, the observed changes filter and gain were able to reproduce the smaller change in output correlations relative to input correlations (B, dark gray bars). (C) Excess correlation computed by comparing responses to different natural movies directly. Our natural stimulus ensemble was composed of movies taken in three outdoor settings. During an experiment, movies from these scenes were interspersed. Here we see that output correlation are similar for all three; thus, in the main text we group all natural movie data together. (TIF) [file pcbi.1003344.s004.tif]

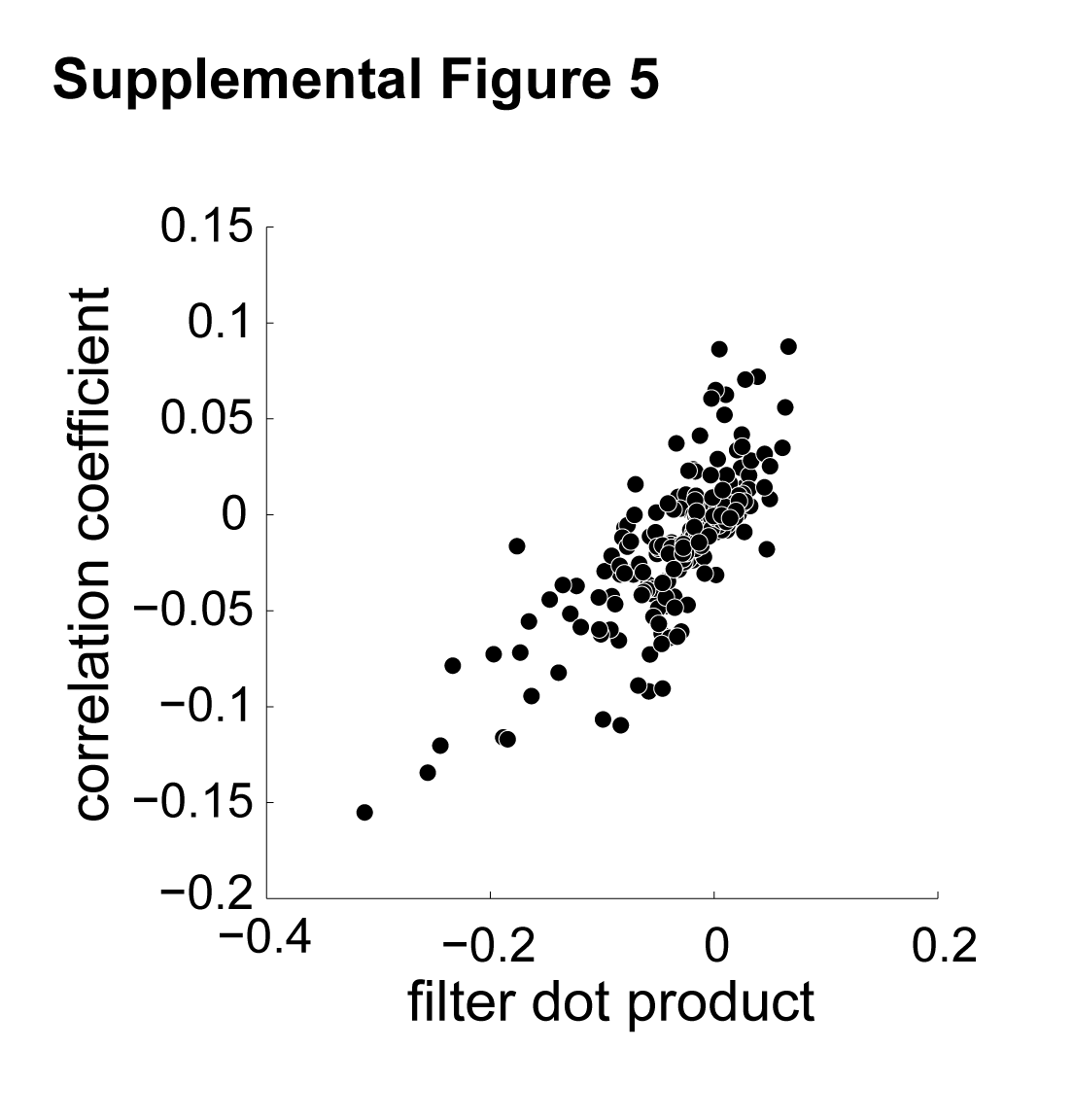

Supplement: Figure S5 — Dependence of correlation on linear filter overlap for opposite-polarity cell pairs. Correlation coefficient between opposite-polarity cell pairs recorded under a white noise stimulus. The dot product of the two cells' spatiotemporal linear filters is used as a measure of the overlap between areas with the same polarity; a pair of cells whose receptive fields are identical in shape but have opposite polarity will have a strong negative filter dot product. The fact that positive correlation between opposite-polarity pairs' spike trains is strongly associated with positive filter dot products may be due to one cell's receptive field center lying in the other's surround or to differences in the temporal filtering of the two cells such that the RF centers do not overlap temporally. (TIF) [file pcbi.1003344.s005.tif]
