# Supplementary material for: Transformation of Stimulus Correlations by the Retina
Source: PLoS Comput Biol. 2013 Dec 5;9(12):e1003344. doi: 10.1371/journal.pcbi.1003344 (PMC3854086; doi:10.1371/journal.pcbi.1003344)
Supplement: Table S1 — Variants on receptive field analysis method. Adaptation index (AI) in surround strength and center latency measured by variations in our analysis method. Columns labeled “mean,” “std,” and “skew” show the mean, standard deviation, and skewness of the adaptation indices for the indicated analysis; columns labeled “p” show the from of each distribution against the null hypothesis of zero mean. Standard analysis: Receptive fields were analyzed as presented in the main text. Disconnected center: The center was not required to form a contiguous region. Surround threshold: A threshold criterion was used to find the surround so that only pixels positively correlated with the peak surround pixel were included. No mean subtraction: The mean of the frames after each spike was not subtracted from the STRFs. First principal component: The full STRF was collapsed onto a single frame by projecting onto the first principal timecourse rather than the principal timecourse most similar to the peak pixel. Masks from WN: Center and surround regions measured from white noise were used to analyze STRFs from both stimuli. Masks from CN: Center and surround regions measured from spatio-temporal exponentially correlated noise were used to analyze STRFs from both stimuli. (PDF) [file pcbi.1003344.s006.pdf]

Table S1. Variants on receptive field analysis method.

	AI (relative surround strength)				AI (center time to peak)			
	mean	std	p	skew	mean	std	p	skew
Standard analysis	0.01	0.24	.008	0.15	−0.03	0.03	$< .0001$	−0.53
Disconnected center	0.10	0.36	.01	−0.01	−0.03	0.22	.2	−2.81
Surround threshold	0.21	0.25	$< .0001$	−0.40	−0.03	0.03	$< .0001$	−0.59
No mean subtraction	0.12	0.23	$< .0001$	0.08	−0.03	0.03	$< .0001$	−0.72
First principal component	0.07	0.24	.01	0.11	−0.03	0.03	$< .0001$	−0.48
Masks from WN	0.02	0.20	.4	0.04	−0.04	0.04	$< .0001$	−2.19
Masks from CN	−0.04	0.19	.08	−0.07	−0.03	0.03	$< .0001$	−0.45

Adaptation index (AI) in surround strength and center latency measured by variations in our analysis method. Columns labeled "mean," "std," and "skew" show the mean, standard deviation, and skewness of the adaptation indices for the indicated analysis; columns labeled "p" show the p-values from t-tests of each distribution against the null hypothesis of zero mean. **Standard analysis**: Receptive fields were analyzed as presented in the main text. **Disconnected center**: The center was not required to form a contiguous region. **Surround threshold**: A threshold criterion was used to find the surround so that only pixels positively correlated with the peak surround pixel were included. **No mean subtraction**: The mean of the frames after each spike was not subtracted from the STRFs. **First principal component**: The full STRF was collapsed onto a single frame by projecting onto the first principal timecourse rather than the principal timecourse most similar to the peak pixel. **Masks from WN**: Center and surround regions measured from white noise were used to analyze STRFs from both stimuli. **Masks from CN**: Center and surround regions measured from exponentially correlated noise were used to analyze STRFs from both stimuli.
