# Supplementary material for: Transformation of Stimulus Correlations by the Retina
Source: PLoS Comput Biol. 2013 Dec 5;9(12):e1003344. doi: 10.1371/journal.pcbi.1003344 (PMC3854086; doi:10.1371/journal.pcbi.1003344)
Supplement: Text S1 — Dependence of output correlation on gain and firing rate. In the main text we found that gain adaptation contributes to decorrelation in a population of LN neurons. In Text S1, we derive a mathematical relationship between the gain of model LN neurons and the pairwise correlations in the model population. (PDF) [file pcbi.1003344.s007.pdf]

Text S1

Dependence of output correlation on gain and firing rate. In the main text we found that gain adaptation contributes to decorrelation in a population of LN neurons. To gain further insight into why this should be the case, consider a pair of simplified LN neurons with logistic nonlinearity,

$$P_i(spike|s) = 1/\left(1 + \exp\left(-g_i\left(s_i - b_i\right)\right)\right) \tag{1}$$

and small firing probabilities in each time bin. For neuron i, the gain of the model is g_i, the linear filter output is s_i, and b_i is an offset that will be adjusted to fix the average firing rate. Assuming a small firing probability amounts to assuming that $g_i(s_i - b_i) \ll 0$ with high probability. Thus, the exponential term in $P_i(spike|s)$ dominates, and the stimulus-dependent firing probability simplifies to $P_i(spike|s) = \exp(g_i(s_i - b_i))$. The average firing probability of one neuron is now

$$P_i = \langle \exp(g_i(s_i - b_i)) \rangle\,, \tag{2}$$

where the average is over the distribution of the filter output s_i, which can be approximated by the central limit theorem as a zero-mean Gaussian. (A nonzero mean could simply be absorbed into a redefinition of b_i.) Using standard properties of Gaussian integrals, the averaging gives

$$P_i = \exp\left[1/2(g_i\sigma_i)^2 - g_i b_i\right], \tag{3}$$

where σ_i is the standard deviation of s_i. Note that, by the low firing probability assumption, $P_1 \ll 1$ and $P_2 \ll 1$.

The average probability of simultaneous firing of two neurons is then given by

$$P_{12} = \langle \exp(g_1(s_1 - b_1)) \exp(g_2(s_2 - b_2)) \rangle \tag{4}$$

$$= \langle \exp(g_1 s_1 + g_2 s_2 - g_1 b_1 - g_2 b_2) \rangle\,. \tag{5}$$

Assuming that the filter outputs are jointly Gaussian with correlation ρ_s, the variance of $g_1 s_1 + g_2 s_2$ is

$(g_1\sigma_1)^2 + (g_2\sigma_2)^2 + 2(g_1\sigma_1)\rho_s(g_2\sigma_2)$. The expectation can therefore be computed as

$$P_{12} = \exp\left[1/2((g_1\sigma_1)^2 + (g_2\sigma_2)^2 + 2(g_1\sigma_1)\rho_s(g_2\sigma_2)) - g_1b_1 - g_2b_2\right] \tag{6}$$

$$= P_1P_2 \exp\left[(g_1\sigma_1)\rho_s(g_2\sigma_2)\right]. \tag{7}$$

The variance in firing of each neuron is $P_i(1 - P_i)$, and the covariance between the two is $P_{12} - P_1P_2$. The correlation coefficient of the two spike trains is then given by

$$\rho = \frac{P_{12} - P_1P_2}{\sqrt{P_1(1 - P_1)P_2(1 - P_2)}}, \tag{8}$$

which, using the above result for P_{12} and taking the limit of small P_i, simplifies to

$$\rho = \sqrt{P_1P_2}\left(e^{(g_1\sigma_1)\rho_s(g_2\sigma_2)} - 1\right). \tag{9}$$

Thus there are three ways to reduce output correlations in this simple model: lower the overall firing rates, decrease ρ_s by filter adaptation, or lower the rescaled gains $g_i\sigma_i$.
