# Supplementary material for: Transformation of Stimulus Correlations by the Retina
Source: PLoS Comput Biol. 2013 Dec 5;9(12):e1003344. doi: 10.1371/journal.pcbi.1003344 (PMC3854086; doi:10.1371/journal.pcbi.1003344)
Supplement: Text S2 — Tests of robustness. To verify that our receptive field results were not an artifact of our specific analysis method, we repeated the analysis with a number of modifications. In Text S2, we report the results of these analyses. (PDF) [file pcbi.1003344.s008.pdf]

Text S2

Tests of robustness. In our experiments, we alternated white noise and correlated stimuli. The retina is known to adapt to a variety of stimulus features on timescales ranging from hundreds of milliseconds to a few seconds. Thus, it is possible that estimating receptive fields using the entire trials confounds different states of adaptation. To control for this possibility, we tested that our analyses were robust to leaving out the first 10 seconds of each trial.

In addition, we also varied the details of the analysis method, as summarized in Table S1. The analysis method presented in the main text was used because it gave the most robustly unbiased results in our simulations, but we also wanted to verify that our results did not change dramatically with slightly different methods. We first considered the possibility that we were including too many pixels in the surround. To address this, we repeated our analysis but placed a threshold criterion on the surround so that only pixels positively correlated with the peak surround pixel were included. Including only these pixels, center latency was still shorter for correlated noise than for white noise (Fig. S1C; "Surround threshold" in Table S1), while the increase in surround strength became more robust than in the original analysis (Fig. S1D). We then tested whether requiring the center to be contiguous was too stringent. Removing this criterion did not change our overall results, although the center time to peak statistics are skewed by a few outliers (Fig. S1E, F; "Disconnected center").

When we computed STRFs, we included three frames after every spike so that we could measure any baseline offset in the estimated STRFs. We generally subtracted the mean of these three frames from each STRF, but skipping this step did not affect our results ("No mean subtraction"). As an additional check, we collapsed the full STRF into a single frame by projecting onto the first principal timecourse rather than the principal timecourse most similar to the peak pixel. Making this change does not affect our results ("First principal component"). (We obtained similar results when we chose the principal timecourse corresponding to the peak surround pixel rather than the peak center pixel.)

To investigate whether the changes we measured in receptive fields came from a change in the size or location of the receptive field center and surround or from a change in the receptive field strength at individual points in space, we repeated our standard analysis with the same masks for both stimuli. That is, for each cell we first found the center region based on the STRF measured from white noise and then computed the time courses of this region under each stimulus condition from the full STRFs. The

surround time courses were computed similarly ("Masks from white noise"). We then did the reverse, finding the center and surround regions from correlated noise STRFs and applying them to both stimuli ("Masks from correlated noise"). In either case, the center latencies were still larger for white noise, indicating that the time courses of individual pixels differ when stimulus correlations change. On the other hand, the relative surround strength adaptation indices were centered around zero when masks were kept fixed. Thus, any changes in surround strength observed in our main analysis were likely due to a subset of pixels switching from center to surround or vice versa.
